# Supplementary material for: Genetic compatibility and ecological connectivity drive the dissemination of antibiotic resistance genes
Source: Nat Commun. 2025 Mar 16;16:2595. doi: 10.1038/s41467-025-57825-3 (PMC11911422; doi:10.1038/s41467-025-57825-3)
Supplement: Supplementary file 1 — Supplementary Information [file 41467_2025_57825_MOESM1_ESM.pdf]

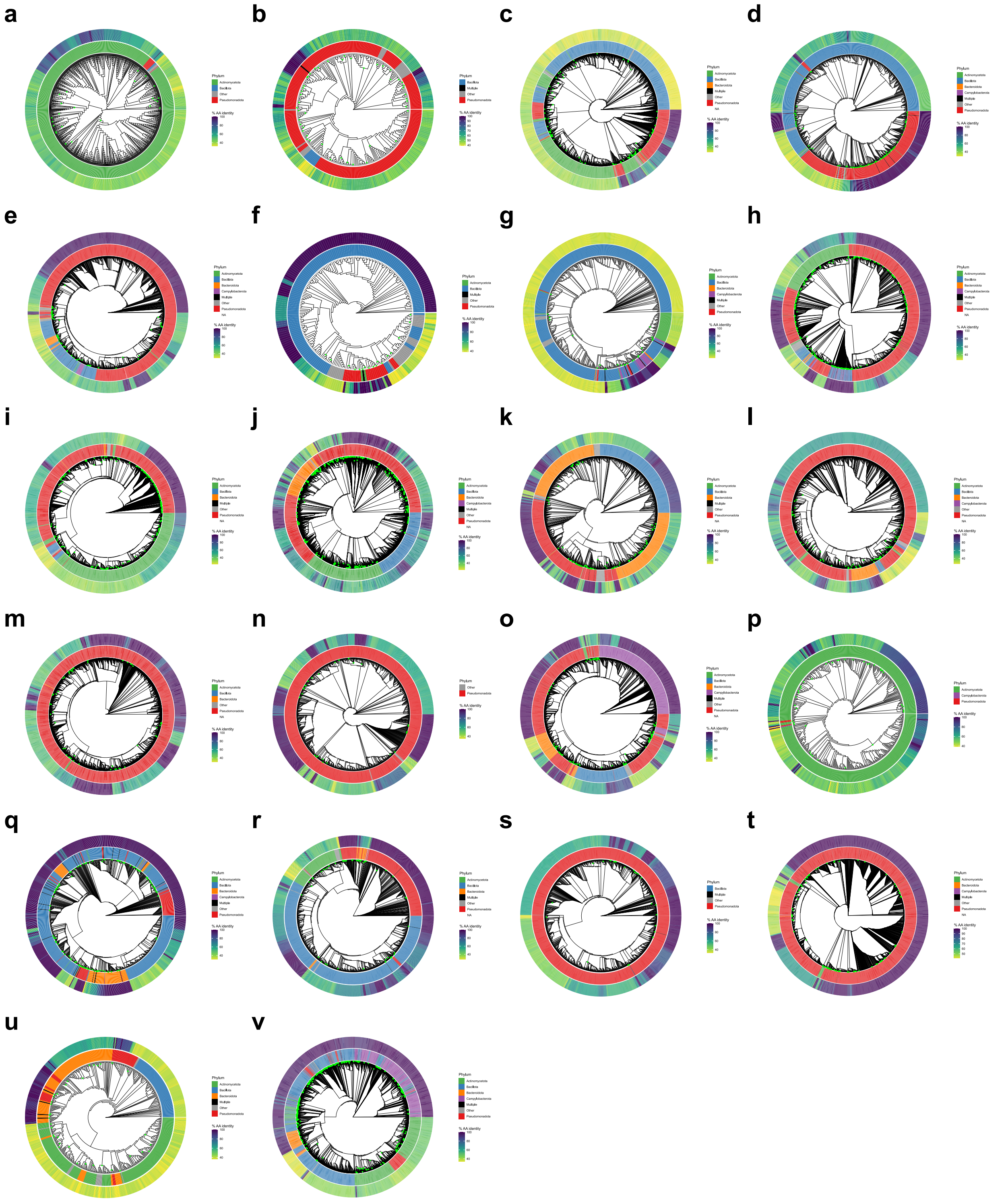

Supplementary Fig. 1 Phylogenetic tree created from unique protein sequences predicted by fARGene, using each of the 22 available gene models. Predicted horizontally transferred antibiotic resistance genes are marked as green dots in the tree. The inner circle shows the phylum to which the bacteria represented by each leaf belong, and the outer circle shows the similarity (% amino acid identity) between each protein and its closest homolog found in the CARD database. **a** Aminoglycoside model A (AAC(2')), **b** Aminoglycoside model B (AAC(3) class 1), **c** Aminoglycoside model C (AAC(3) class 2), **d** Aminoglycoside model D (AAC(6') class 1), **e** Aminoglycoside model E

(AAC(6') class 2), **f** Aminoglycoside model F (AAC(6') class 3), **g** Aminoglycoside model G (APH(2'')), **h** Aminoglycoside model H (APH(3')), **i** Aminoglycoside model I (APH(6)), **j** Class A beta-lactamases, **k** Class B1/B2 beta-lactamases, **l** Class B3 beta-lactamases, **m** Class C beta-lactamases, **n** Class D1 beta-lactamases, **o** Class D2 beta-lactamases, **p** Erm 23S rRNA methyltransferase type A, **q** Erm 23S rRNA methyltransferase type F, **r** Mph 2'-macrolide phosphotransferases, **s** Quinolone resistance genes (Qnr), **t** Tetracycline efflux pumps, **u** Tetracycline inactivation enzymes, **v** Tetracycline ribosomal protection genes (RPGs).

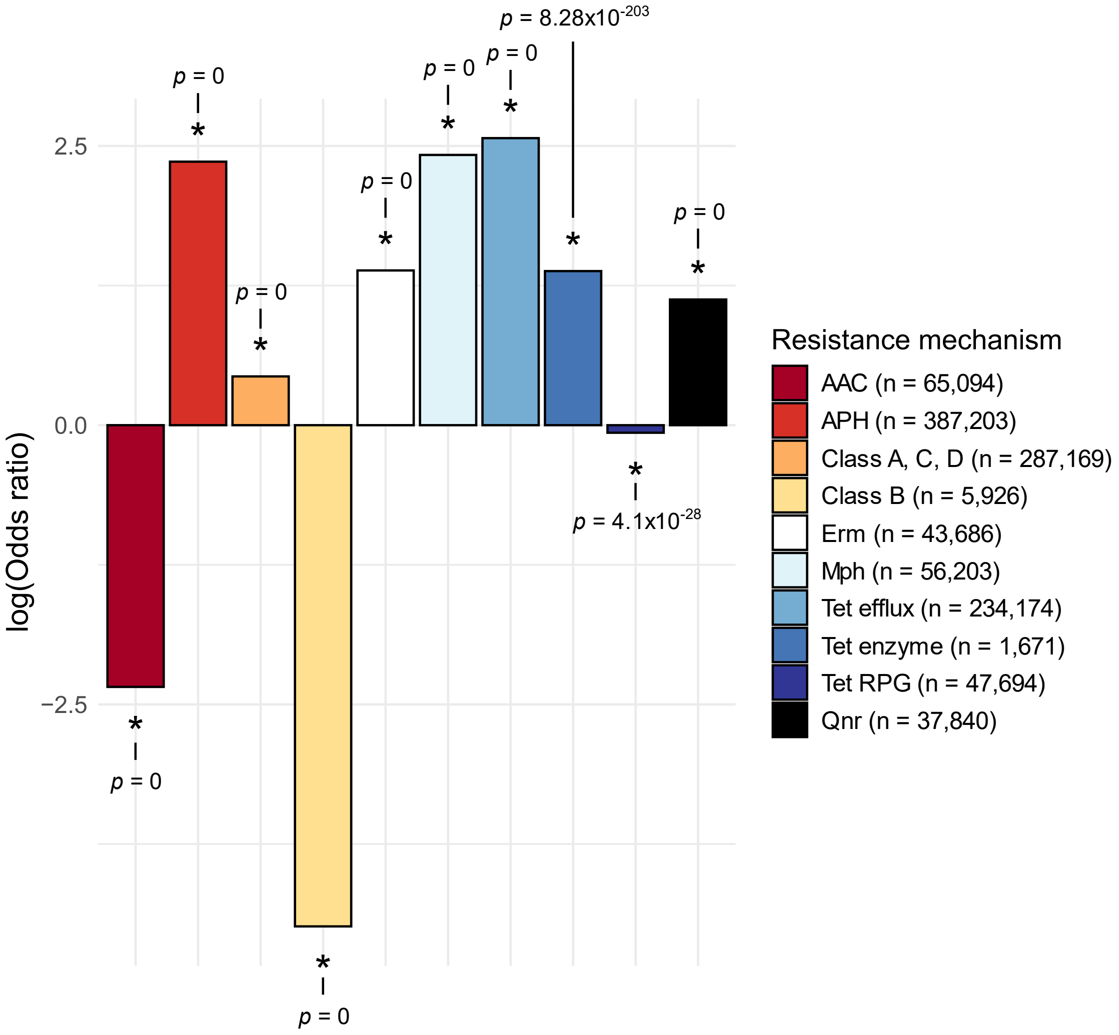

Supplementary Fig. 2 Enrichment analysis of predicted antibiotic resistance genes involved in identified horizontal transfers. The genes have been stratified based on encoded resistance mechanism, including aminoglycoside acetyltransferases (AAC), aminoglycoside phosphotransferases (APH), class A, C, D beta-lactamases, class B beta-lactamases, Erm 23S rRNA methyltransferases, Mph 2'-macrolide phosphotransferases, tetracycline efflux pumps (Tet efflux), tetracycline inactivating enzymes (Tet enzyme), tetracycline ribosomal protection genes (Tet RPG), and quinolone resistance genes (Qnr). The ratios and their significance were calculated using Fisher's exact test. *$P < 0.01$. Source data are provided as a Source Data file.

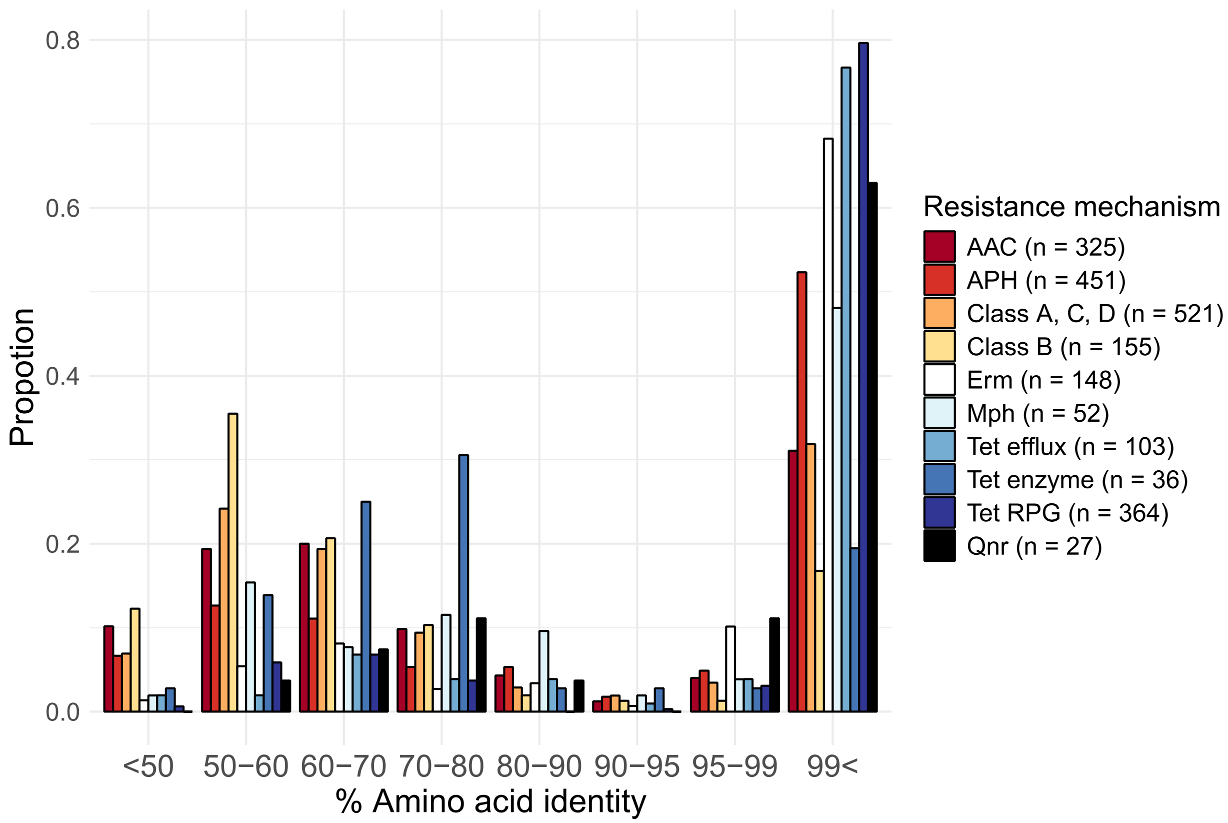

Supplementary Fig. 3 Maximum similarity (% amino acid identity) observed between the antibiotic resistance genes carried by species from different taxonomic orders in the observed horizontal transfers. Here, each leaf or node in the tree where a transfer was observed is counted only once. The observations are stratified based on the resistance mechanism that the transferred gene encodes, including aminoglycoside acetyltransferases (AAC), aminoglycoside phosphotransferases (APH), class A, C, D beta-lactamases, class B beta-lactamases, Erm 23S rRNA methyltransferases, Mph 2'-macrolide phosphotransferases, tetracycline efflux pumps (Tet efflux), tetracycline inactivating enzymes (Tet enzyme), tetracycline ribosomal protection genes (Tet RPG), and quinolone resistance genes (Qnr). For each resistance mechanism, the number of observed transfers is included in the legend. Source data are provided as a Source Data file.

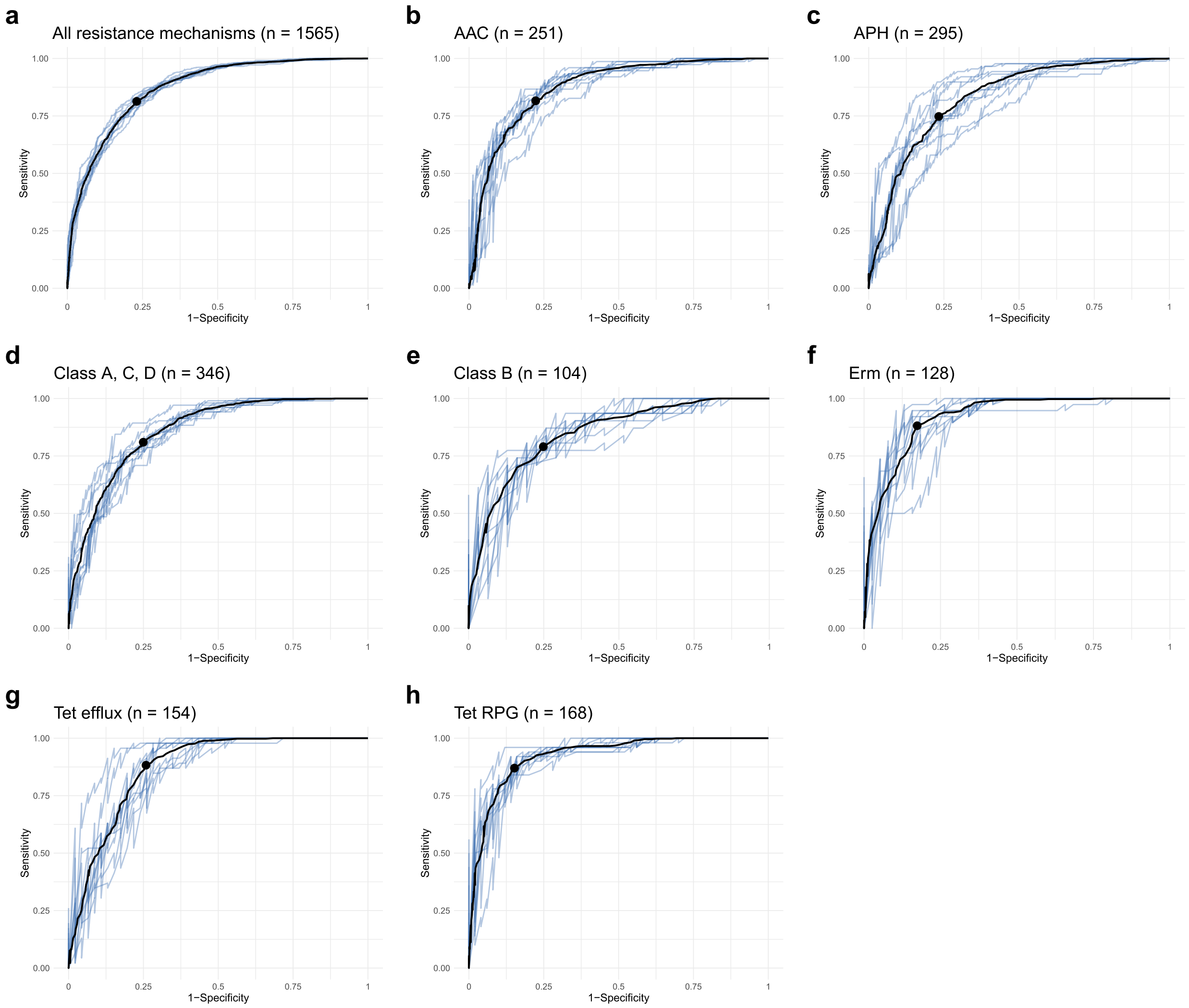

Supplementary Fig. 4 Reciever operating characteristic curves produced from predictions on test data by random forest classifiers. The models were trained on horizontal transfers representing **a** all included resistance mechanisms, **b** AAC aminoglycoside acetyltransferases, **c** APH aminoglycoside phosphotransferases, **d** Class A, C, D beta-lactamases, **e** Class B beta-lactamases, **f** Erm 23S rRNA methyltransferases, **g** Tetracycline efflux pumps (Tet efflux), **h** Tetracycline ribosomal protection genes (Tet RPG). A point is placed on each curve representing the observed optimal performance. The number of observed transfers making up the training + test data for each model is included in the titles. An equal number of randomized transfers were used for each model as the negative dataset. Source data are provided as a Source Data file.

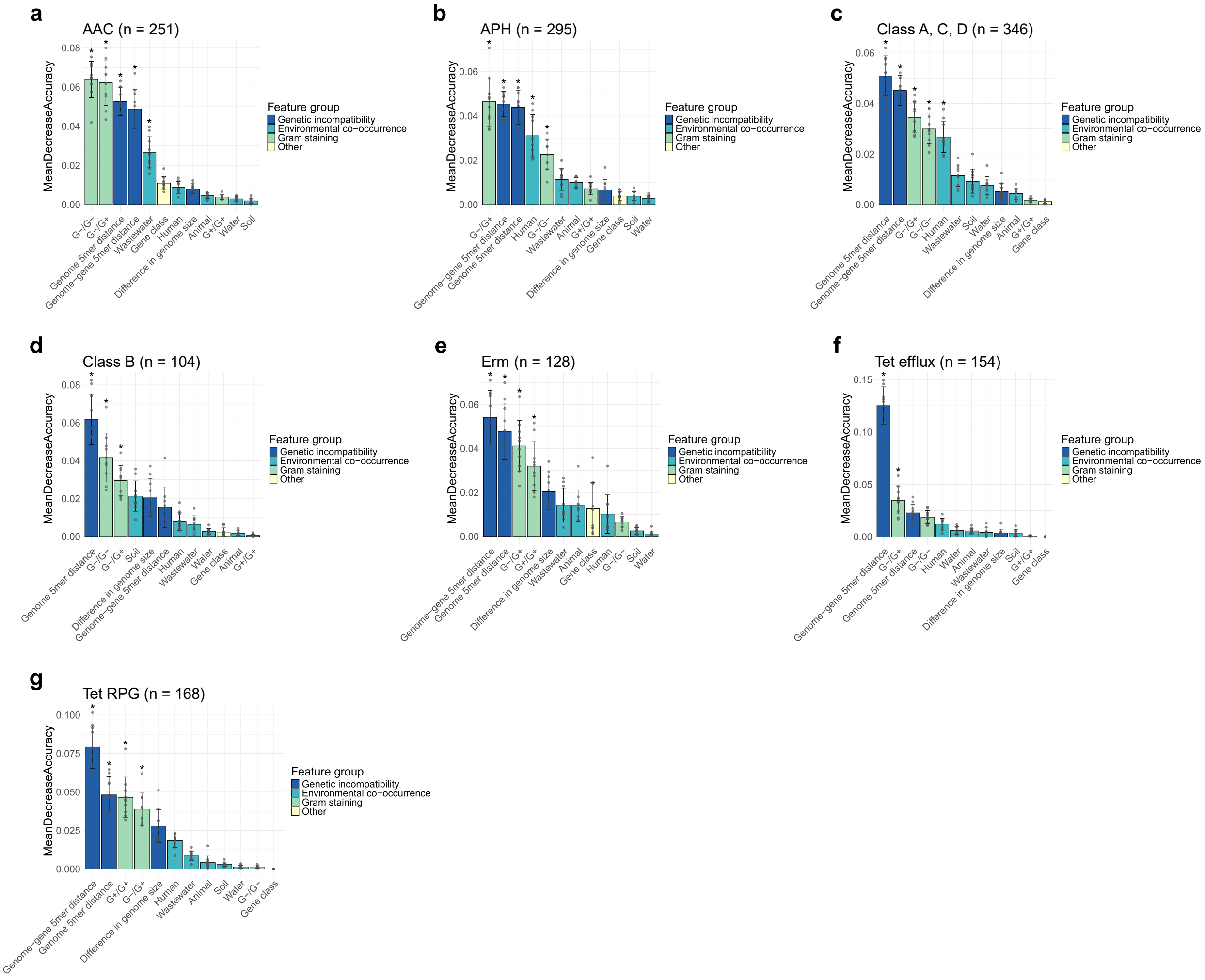

Supplementary Fig. 5 Permutation importance of all features included in the resistance mechanism-specific random forest classifiers. In all instances, the bars show the mean +/- SD of the importance of each factor to the accuracy of the model (MeanDecreaseAccuracy) over ten iterations. Permutation tests were used to generate a p-value for each factor and iteration. All individual p-values are available in the Source Data. *P<0.01 across all model iterations. Features are ordered according to their overall contribution to the accuracy of the model trained to predict horizontal transfers of **a** aminoglycoside acetyltransferases (AAC), **b** aminoglycoside phosphotransferases (APH), **c** class A, C, D beta-lactamases, **d** class B beta-lactamases, **e** Erm 23S rRNA methyltransferases, **f** tetracycline efflux pumps (Tet efflux), and **g** tetracycline ribosomal protection genes (Tet RPG). The number of observed transfers making up the training + test data for each model is included in the titles. An equal number of randomized transfers were used for each model as the negative dataset. Source data are provided as a Source Data file.

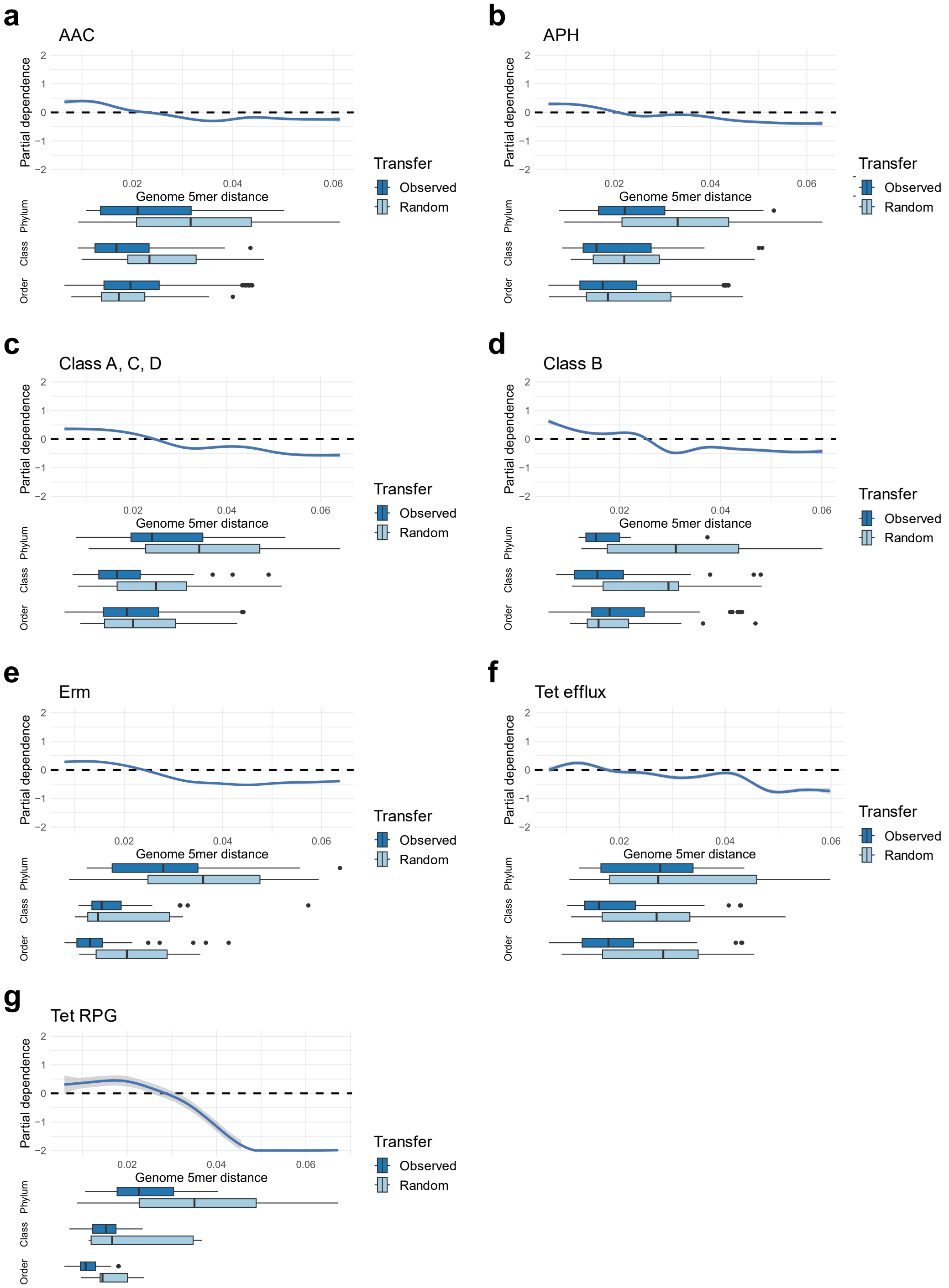

Supplementary Fig. 6 Relative contribution of the genome 5mer distance to the classification of horizontally spread antibiotic resistance genes for the mechanism-specific random forest models. The distributions of values seen for the observed and the randomized transfers at different taxonomic levels is visualized as boxplots below the main graph. Here, the centerline, box limits, and whiskers indicate the median, interquartile range, and 1,5 × interquartile range, respectively. The minima and maxima represent the minimum and maximum values observed. **a** Aminoglycoside acetyltransferases (AAC). **b** Aminoglycoside phosphotransferases (APH). **c** Class A, C, D beta-lactamases. **d** Class B beta-lactamases. **e** Erm 23S rRNA methyltransferases. **f** Tetracycline efflux pumps (Tet efflux). **g** Tetracycline ribosomal protection genes (Tet RPG). Source data are provided as a Source Data file.

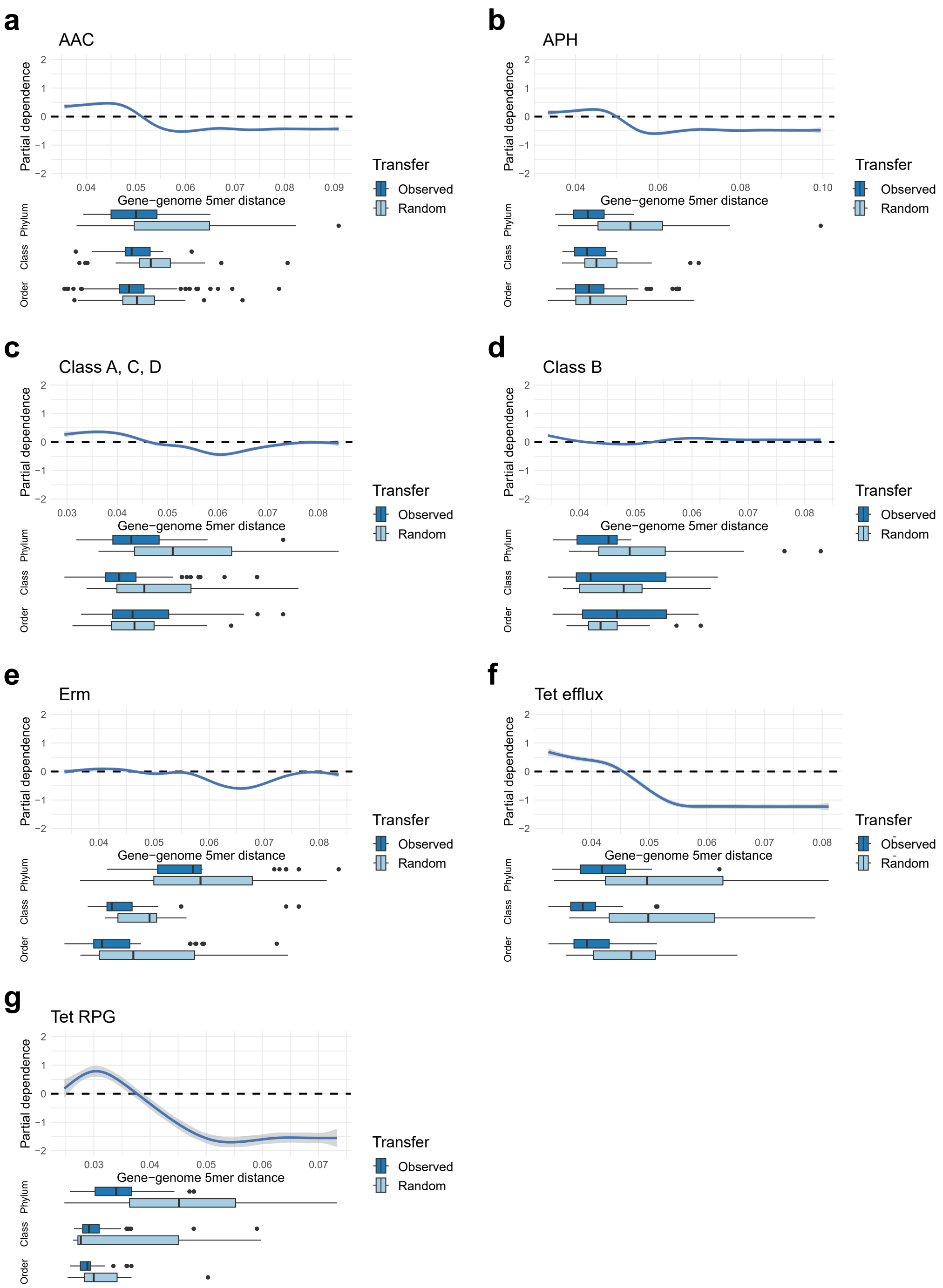

Supplementary Fig. 7 Relative contribution of the gene-genome 5mer distance to the classification of horizontally spread antibiotic resistance genes for the mechanism-specific random forest models. The distributions of values seen for the observed and the randomized transfers at different taxonomic levels are visualized as boxplots below the main graph. Here, the centerline, box limits, and whiskers indicate the median, interquartile range, and 1,5 × interquartile range, respectively. The minima and maxima represent the minimum and maximum values observed. **a** Aminoglycoside acetyltransferases (AAC). **b** Aminoglycoside phosphotransferases (APH). **c** Class A, C, D beta-lactamases. **d** Class B beta-lactamases. **e** Erm 23S rRNA methyltransferases. **f** Tetracycline efflux pumps (Tet efflux). **g** Tetracycline ribosomal protection genes (Tet RPG). Source data are provided as a Source Data file.

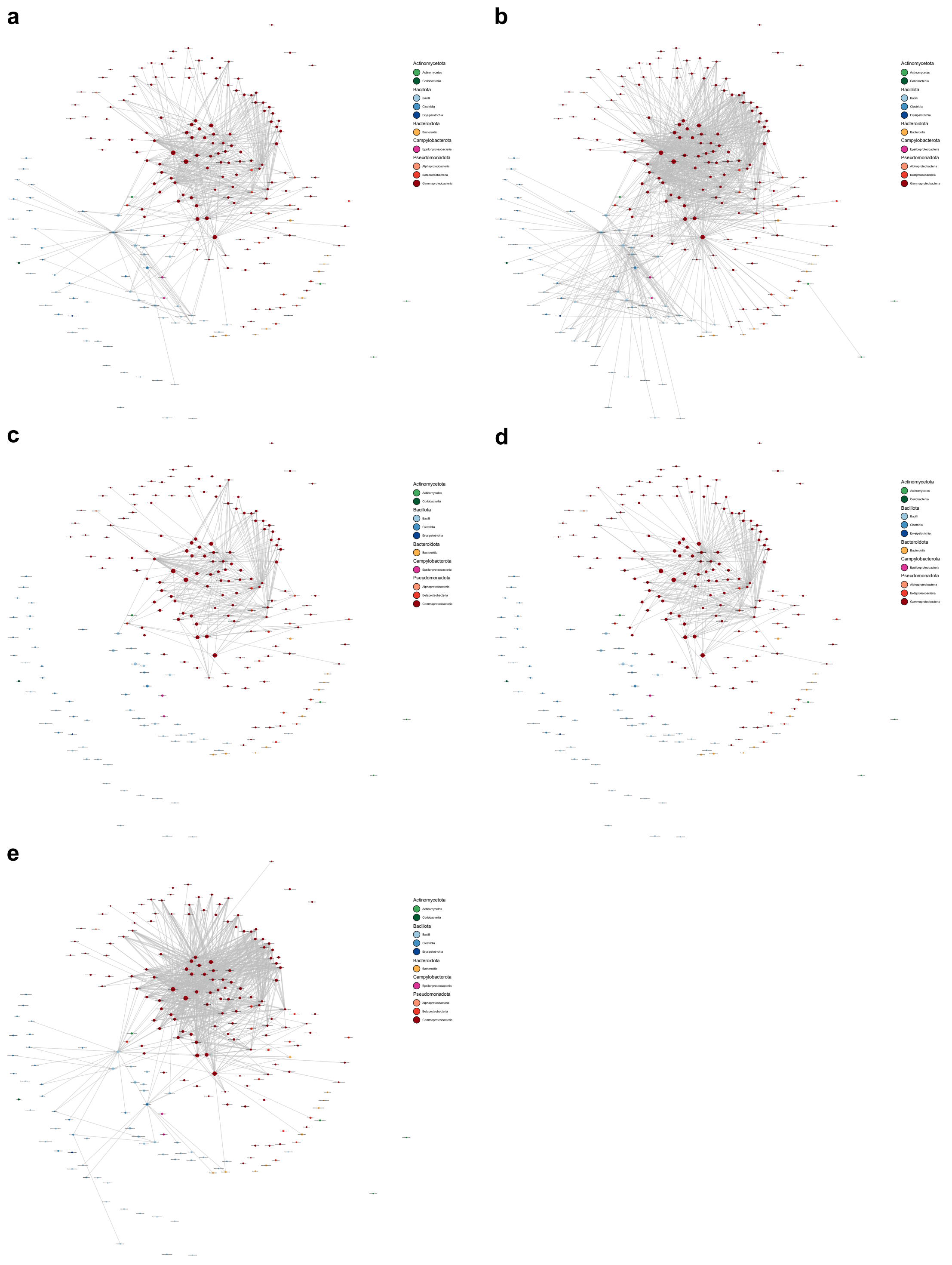

Supplementary Fig. 8 Co-occurrence of promiscuous bacterial species in animal, human, soil, water, and wastewater microbiomes. Each node represents a species, the size is proportional to the total number of inferred interactions associated with that species. Edges are drawn between species with a ≥ order-level distance, between which horizontal transfer was observed ≥ 5 times. Edge thickness indicates the maximal estimated co-occurrence of two species included in each taxon in **a** Animal samples (*n* = 4,376), **b** Human samples (*n* = 3,220), **c** Soil samples (*n* = 4,137), **d** Water samples (*n* = 7,898), and **e** Wastewater samples (*n* = 1,185). If co-occurrence was measurable in less than 1% of the corresponding samples, no edge is drawn. Source data are provided as a Source Data file.

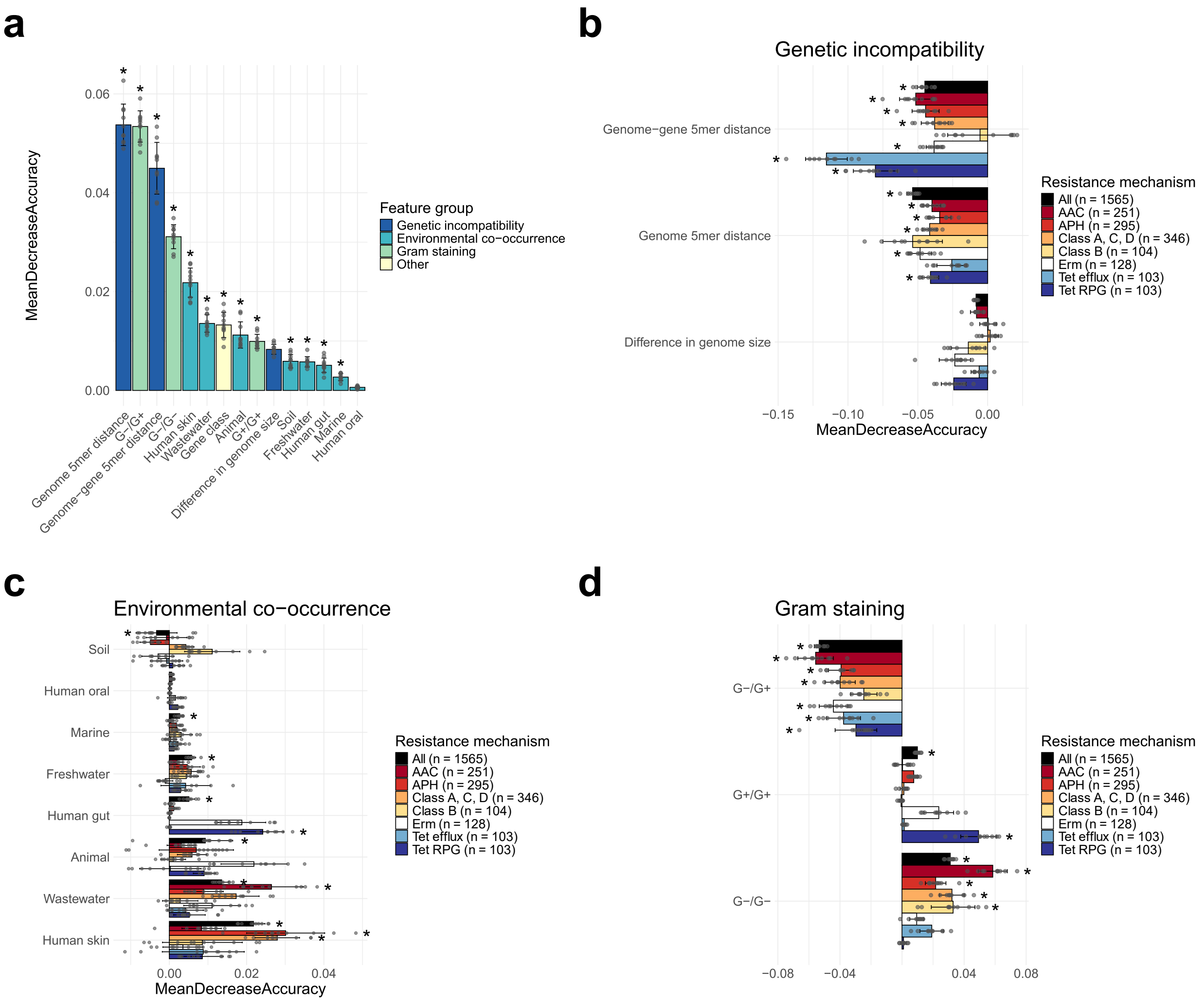

Supplementary Fig. 9. Relative importance of factors from models using more specific co-occurrence variables. In all instances, the bars show the mean +/- SD of the importance of each factor to the accuracy of the model (MeanDecreaseAccuracy) over ten iterations. Permutation tests were used to generate a *p*-value for each factor and iteration. All individual *p*-values are available in the Source Data. *P<0.01 across all model iterations. **a** The importance of the factors included in the general random forest model, based on all observed transfers (*n* = 1,565), ordered according to their overall contribution to the accuracy of the model. **b–d** The mean importance of each factor group (genetic incompatibility, environmental co-occurrence, and Gram staining, respectively) for each random forest models over ten iterations. In addition to the general model (All), seven models specific to different resistance mechanisms, including aminoglycoside acetyltransferases (AAC), aminoglycoside phosphotransferases (APH), class A, C, D beta-lactamases, class B beta-lactamases, Erm 23S rRNA methyltransferases, tetracycline efflux pumps (Tet efflux), and tetracycline ribosomal protection genes (Tet RPG) are included. Signs have been added to show whether an increased value of the variable is generally indicative of horizontally spread resistance genes (+) or not (–) based on partial dependence analysis. The number of observed transfers making up the training + test data for each model is included in the legends. An equal number of randomized transfers were used for each model as the negative dataset. Source data are provided as a Source Data file.

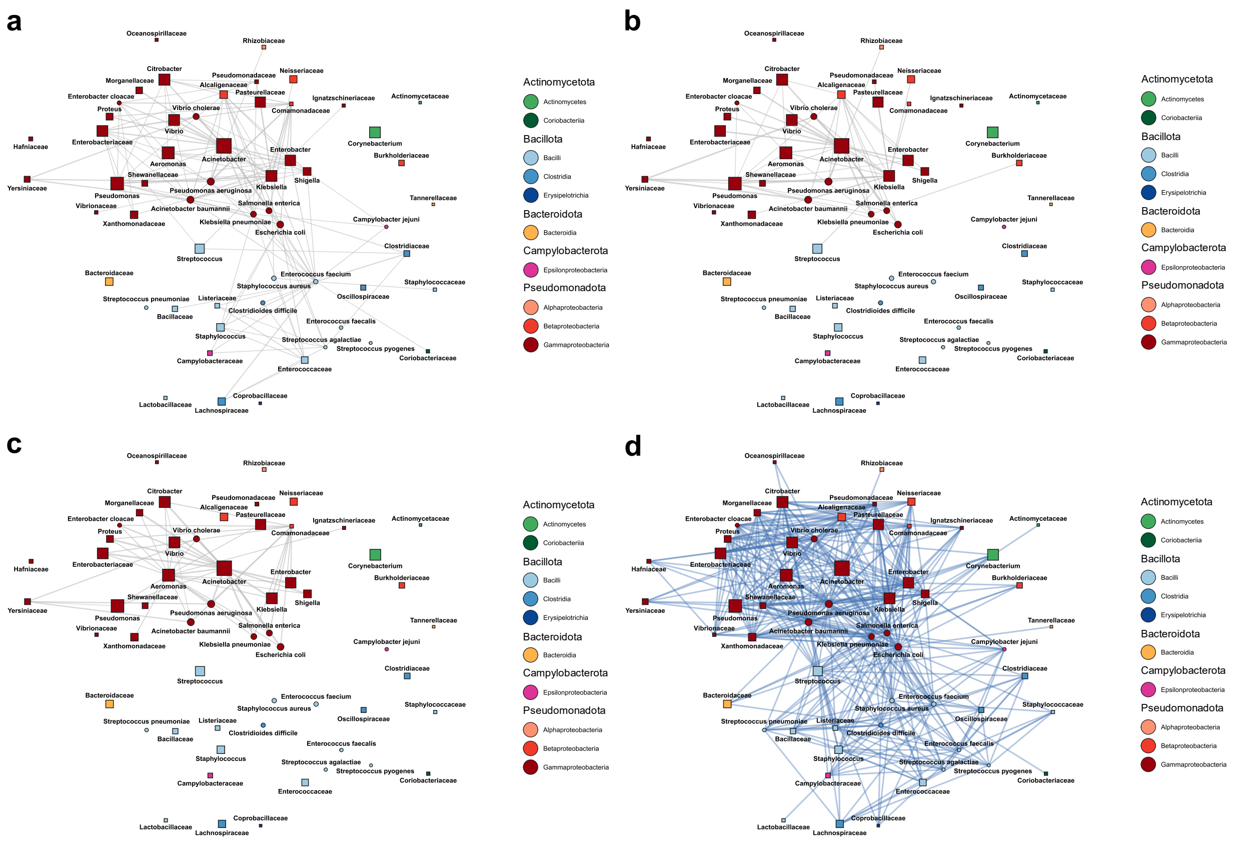

Supplementary Fig. 10 Co-occurrence of promiscuous bacterial taxa in animal, soil, and water microbiomes. In the networks, each node represents a taxon on either species, genus, or family level, which was aggregated such that individual nodes on a lower level are not part of the corresponding higher-level node(s). For each node, the size is proportional to the total number of inferred interactions associated with that taxon, and the shape indicates if the node represents a species (circle) or a higher-level taxon (square). Edges are drawn between taxa with a ≥ order-level distance, between which horizontal transfer was observed ≥ 5 times. Edge thickness indicates the maximal estimated co-occurrence of two species included in each taxon in **a** Animal samples (*n* = 4,376), **b** Soil samples (*n* = 4,137), and **c** Water samples (*n* = 7,898). If co-occurrence was measurable in < 1% of the corresponding samples, no edge is drawn. Panel **d** shows all frequently observed interactions (≥ 5 transfers) between bacterial taxa without including any information from the metagenomic samples. Since no estimated co-occurrence is shown, all edges are drawn with equal thickness and blue color to differentiate them. Source data are provided as a Source Data file.

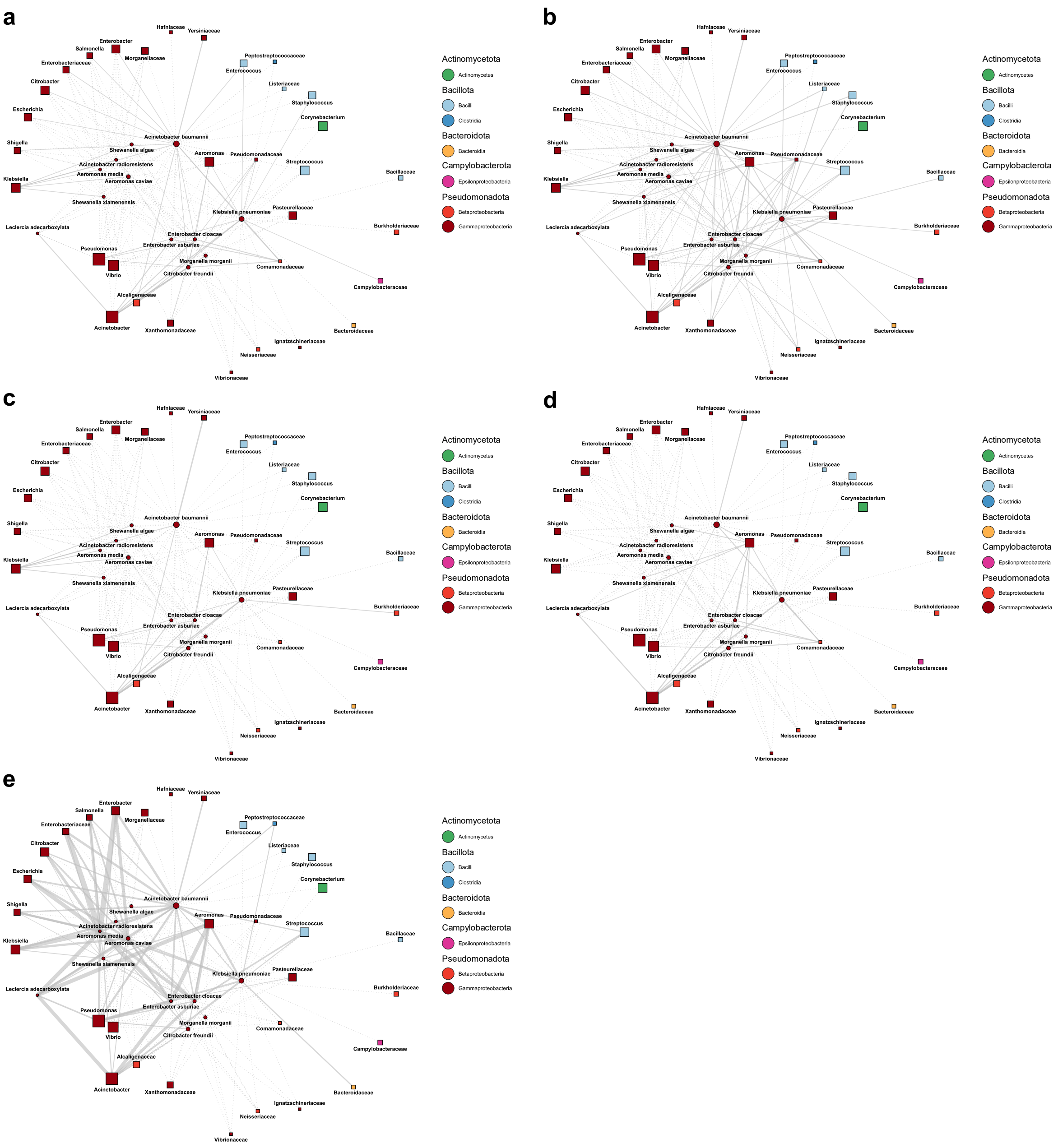

Supplementary Fig. 11 Networks displaying the observed gene transfers between species that have been identified as recent origins of mobile antibiotic resistance genes and other bacterial taxa, as well as their co-occurrence in different environments. Each node represents either a species that was identified as a likely recent origin more mobile resistance gene(s) in Ebmeyer et al 2021 (https://doi.org/10.1038/s42003-020-01545-5) or a taxon on either genus, or family level, which was aggregated such that individual nodes on a lower level are not part of the corresponding higher-level node(s). Edges are only drawn between identified origin species and other taxa between which horizontal transfer was observed ≥ 5 times. The size of each node is proportional to the total number of inferred interactions associated with that taxon, and the shape indicates if the node represents an origin species (circle) or a higher-level taxon (square). Edge thickness indicates the maximal estimated co-occurrence of two species included in each taxon in in **a** Animal samples (*n* = 4,376), **b** Human samples (*n* = 3,220), **c** Soil samples (*n* = 4,137), **d** Water samples (*n* = 7,898), and **e** Wastewater samples (*n* = 1,185). If co-occurrence was measurable in less than 1% of the corresponding samples, the connection is represented as a dashed line. Source data are provided as a Source Data file.

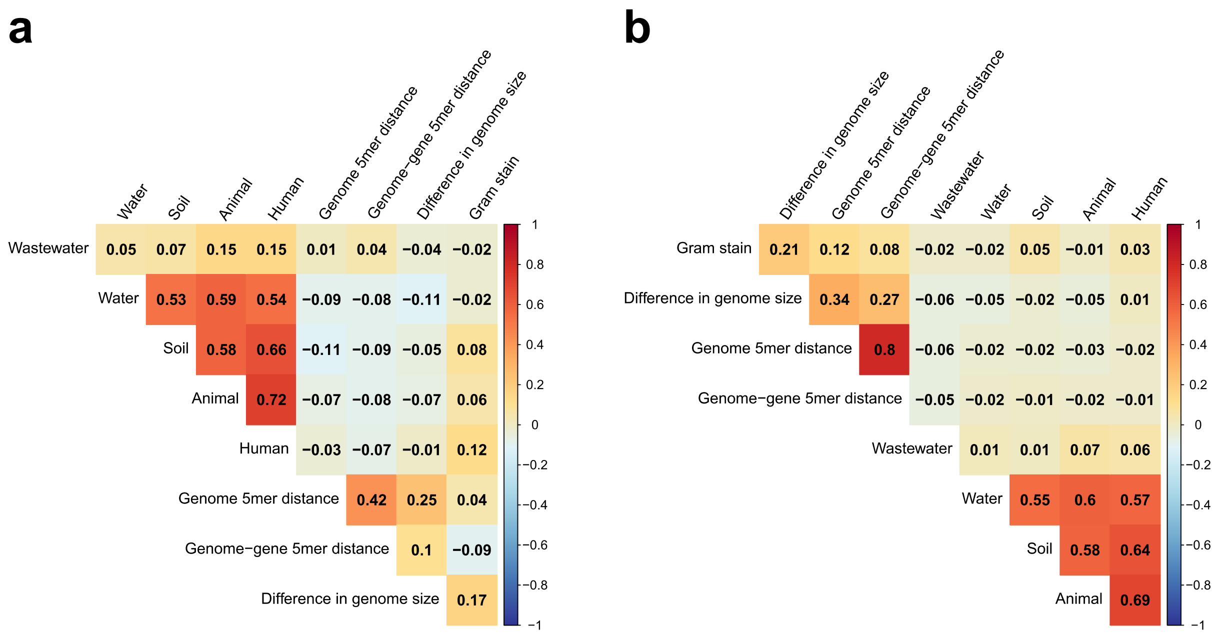

Supplementary Fig. 12 Spearman's correlation of the features used to train random forest models. a Correlations seen for observed transfers. **b** Correlations seen for randomized transfers. Source data are provided as a Source Data file.

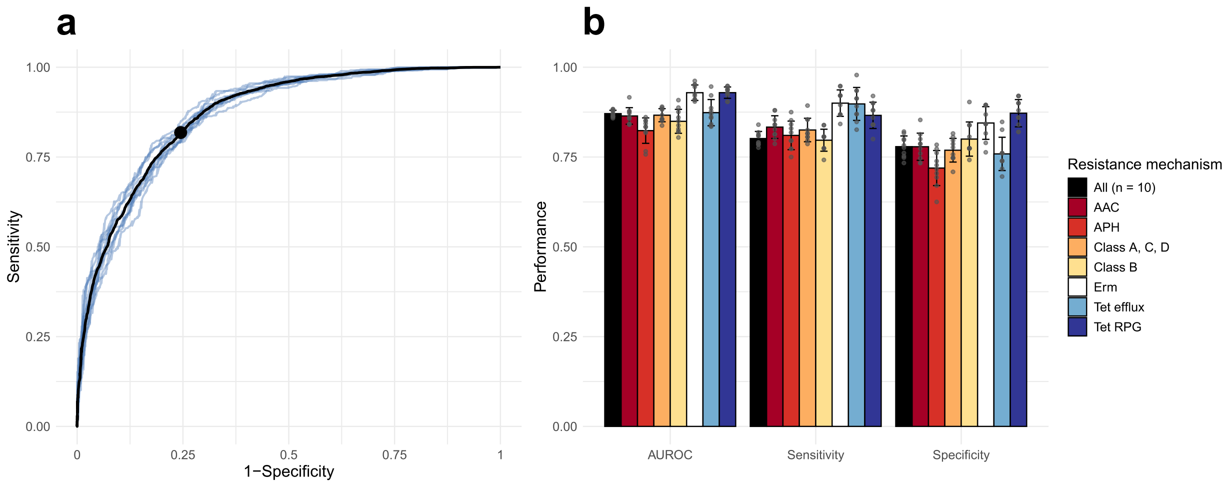

Supplementary Fig. 13. Performance of models using more specific co-occurrence variables. a Receiver operating characteristic (ROC) curves produced from predictions on test data by random forest models trained on horizontal transfers representing all included resistance mechanisms, over ten iterations. Each model was built using variables representing the genetic incompatibility, environmental co-occurrence, and cell wall composition of the bacteria involved in each transfer. The black line represents the mean of the produced ROC curves. The point represents the mean optimal performance (the point closest to a sensitivity and specificity of 1). **b** Area under the ROC curve (AUROC), sensitivity, and specificity observed for predictions on test data using random forest models representing different resistance mechanisms with enough data present (>100 transfers observed). In addition to the general model (All), this included aminoglycoside acetyltransferases (AAC), aminoglycoside phosphotransferases (APH), class A, C, D beta-lactamases, class B beta-lactamases, Erm 23S rRNA methyltransferases, tetracycline efflux pumps (Tet efflux), and tetracycline ribosomal protection genes (Tet RPG). The bars show the mean +/- SD of the observed metrics over ten iterations. Source data are provided as a Source Data file.

Supplementary Table 1. Summary of predicted antibiotic resistance genes (ARGs) and horizontally spread ARGs

Gene type	Predicted ARGs [unique protein sequences]	Detected horizontal transfers [unique points in the tree]
**Aminoglycoside**		
AAC(2’)	3,474 [879]	24 [24]
AAC(3) class 1	2,467 [295]	62 [46]
AAC(3) class 2	114,495 [3,853]	163 [98]
AAC(6’) class 1	22,520 [1,081]	258 [83]
AAC(6’) class 2	471,068 [2,512]	69 [58]
AAC(6’) class 3	22,164 [227]	31 [17]
APH(2’’)	3,729 [568]	33 [24]
APH(3’)	252,441 [3,368]	1,198 [241]
APH(6)	198,264 [5,010]	648 [186]
**Beta-lactam**		
Class A	291,085 [12,399]	1,196 [353]
Class B1/B2	12,713 [2,017]	130 [85]
Class B3	461,461 [3,255]	70 [70]
Class C	90,543 [7,329]	60 [53]
Class D1	40,392 [1,536]	73 [27]
Class D2	126,394 [3,274]	163 [89]
**Macrolide**		
Erm type A	1,433 [461]	53 [39]
Erm type F	56,706 [938]	310 [109]
Mph	62,924 [1,551]	140 [52]
**Quinolone**		
Qnr	53,980 [1,670]	133 [27]
**Tetracycline**		
Tet efflux	262,452 [3,161]	700 [103]
Tet enzyme	2,212 [448]	57 [36]
Tet RPG	113,085 [4,964]	705 [324]
**Total**	**2,666,002 [60,773]**	**6,276 [2,144]**
