# Supplementary material for: Genetic compatibility and ecological connectivity drive the dissemination of antibiotic resistance genes
Source: Nat Commun. 2025 Mar 16;16:2595. doi: 10.1038/s41467-025-57825-3 (PMC11911422; doi:10.1038/s41467-025-57825-3)
Supplement: Supplementary file 2 — Description of Additional Supplementary Files [file 41467_2025_57825_MOESM2_ESM.docx]

Description of Additional Supplementary Files

**File Name:** Supplementary Data 1

**Description:** Accession IDs and Sample IDs of the genomes and metagenomes analyzed in this study.

**File Name:** Supplementary Data 2

**Description:** Accession IDs of genomes and the operational taxonomic units to which they were matched.
