# Supplementary material for: Genetic compatibility and ecological connectivity drive the dissemination of antibiotic resistance genes
Source: Nat Commun. 2025 Mar 16;16:2595. doi: 10.1038/s41467-025-57825-3 (PMC11911422; doi:10.1038/s41467-025-57825-3)
Supplement: Supplementary file 5 — Reporting Summary [file 41467_2025_57825_MOESM5_ESM.pdf]

nature portfolio

Corresponding author(s): Erik Kristiansson

Last updated by author(s): Feb 21, 2025

Reporting Summary

Nature Portfolio wishes to improve the reproducibility of the work that we publish. This form provides structure for consistency and transparency in reporting. For further information on Nature Portfolio policies, see our Editorial Policies and the Editorial Policy Checklist.

Statistics

For all statistical analyses, confirm that the following items are present in the figure legend, table legend, main text, or Methods section.

n/a	Confirmed	
☐	☒	The exact sample size (*n*) for each experimental group/condition, given as a discrete number and unit of measurement
☒	☐	A statement on whether measurements were taken from distinct samples or whether the same sample was measured repeatedly
☐	☒	The statistical test(s) used AND whether they are one- or two-sided<br>*Only common tests should be described solely by name; describe more complex techniques in the Methods section.*
☐	☒	A description of all covariates tested
☒	☐	A description of any assumptions or corrections, such as tests of normality and adjustment for multiple comparisons
☐	☒	A full description of the statistical parameters including central tendency (e.g. means) or other basic estimates (e.g. regression coefficient) AND variation (e.g. standard deviation) or associated estimates of uncertainty (e.g. confidence intervals)
☐	☒	For null hypothesis testing, the test statistic (e.g. *F*, *t*, *r*) with confidence intervals, effect sizes, degrees of freedom and *P* value noted<br>*Give P values as exact values whenever suitable.*
☒	☐	For Bayesian analysis, information on the choice of priors and Markov chain Monte Carlo settings
☒	☐	For hierarchical and complex designs, identification of the appropriate level for tests and full reporting of outcomes
☒	☐	Estimates of effect sizes (e.g. Cohen's *d*, Pearson's *r*), indicating how they were calculated

Our web collection on statistics for biologists contains articles on many of the points above.

Software and code

Policy information about availability of computer code

Data collection	No software was used to download data.
Data analysis	Antibiotic resistance genes (ARGs) were predicted in bacterial genomes using fARGene v0.1. Protein sequences were clustered using USEARCH v8.01445, and aligned using mafft v7.458. Phylogenetic trees were created using FastTree v2.1.19, and from these a custom algorithm was used to identify horizontally transferred ARGs. Custom code was implemented to calculate features for the observed transfers. This custom code is available at https://github.com/davidgllund/factors_influencing_HGT_of_ARGs and https://doi.org/10.5281/zenodo.13827913. Phylogenetic trees were visualized using ggtree v2.0.0. The similarity of protein sequences was assessed through alignment using BLAST+ v2.10.1, which was also used to align genomes and operational taxonomic units. To train random forest models and estimate feature importance the R packages randomForest v4.7-1.1, rfPermute v2.5.1, and rfUtilities v2.1-5 were used. The performance of the models was assessed by using the pROC v1.18 R package. Partial dependence analysis was performed using the pdp v0.8.1 R package. Co-occurrence networks were generated and visualized using the R packages igraph v1.4.3 and GGally v2.1.2. The code and files used to produce all of the main and supplementary Figures from the paper are available at https://doi.org/10.5281/zenodo.14901409.

For manuscripts utilizing custom algorithms or software that are central to the research but not yet described in published literature, software must be made available to editors and reviewers. We strongly encourage code deposition in a community repository (e.g. GitHub). See the Nature Portfolio guidelines for submitting code & software for further information.

Data

Policy information about availability of data

All manuscripts must include a data availability statement. This statement should provide the following information, where applicable:

- Accession codes, unique identifiers, or web links for publicly available datasets
- A description of any restrictions on data availability
- For clinical datasets or third party data, please ensure that the statement adheres to our policy

All raw data used for this study have been retrieved from public repositories including NCBI (https://www.ncbi.nlm.nih.gov/), the Earth Microbiome Project (https://earthmicrobiome.org/), and the Global Water Microbiome Consortium (http://gwmc.ou.edu/). Accession numbers of the analyzed genomes and metagenomes are provided in Supplementary Data 1. The intermediary data generated in this study are available via Zenodo at https://doi.org/10.5281/zenodo.1490140971. Source data are provided with this paper.

Research involving human participants, their data, or biological material

Policy information about studies with human participants or human data. See also policy information about sex, gender (identity/presentation), and sexual orientation and race, ethnicity and racism.

Reporting on sex and gender	N/A
Reporting on race, ethnicity, or other socially relevant groupings	N/A
Population characteristics	N/A
Recruitment	N/A
Ethics oversight	N/A

Note that full information on the approval of the study protocol must also be provided in the manuscript.

Field-specific reporting

Please select the one below that is the best fit for your research. If you are not sure, read the appropriate sections before making your selection.

[x] Life sciences [ ] Behavioural & social sciences [ ] Ecological, evolutionary & environmental sciences

For a reference copy of the document with all sections, see nature.com/documents/nr-reporting-summary-flat.pdf

Life sciences study design

All studies must disclose on these points even when the disclosure is negative.

Sample size	The sample size was determined by the number of publicly available bacterial genomes in GenBank. No additional data was collected for this study.
Data exclusions	Bacterial genomes with uncertainties regarding their taxonomic annotation were excluded to ensure the robustness of our results. Otherwise no data was excluded.
Replication	N/A
Randomization	N/A
Blinding	N/A

Reporting for specific materials, systems and methods

We require information from authors about some types of materials, experimental systems and methods used in many studies. Here, indicate whether each material, system or method listed is relevant to your study. If you are not sure if a list item applies to your research, read the appropriate section before selecting a response.

Materials & experimental systems

n/a	Involved in the study
☒	☐ Antibodies
☒	☐ Eukaryotic cell lines
☒	☐ Palaeontology and archaeology
☒	☐ Animals and other organisms
☒	☐ Clinical data
☒	☐ Dual use research of concern
☒	☐ Plants

Methods

n/a	Involved in the study
☒	☐ ChIP-seq
☒	☐ Flow cytometry
☒	☐ MRI-based neuroimaging

Plants

Seed stocks	*Report on the source of all seed stocks or other plant material used. If applicable, state the seed stock centre and catalogue number. If plant specimens were collected from the field, describe the collection location, date and sampling procedures.*
Novel plant genotypes	*Describe the methods by which all novel plant genotypes were produced. This includes those generated by transgenic approaches, gene editing, chemical/radiation-based mutagenesis and hybridization. For transgenic lines, describe the transformation method, the number of independent lines analyzed and the generation upon which experiments were performed. For gene-edited lines, describe the editor used, the endogenous sequence targeted for editing, the targeting guide RNA sequence (if applicable) and how the editor was applied.*
Authentication	*Describe any authentication procedures for each seed stock used or novel genotype generated. Describe any experiments used to assess the effect of a mutation and, where applicable, how potential secondary effects (e.g. second site T-DNA insertions, mosiacism, off-target gene editing) were examined.*
